# Discovering Interdisciplinary Research Based on Neural Networks

**DOI:** 10.3389/fbioe.2022.908733

**Published:** 2022-06-03

**Authors:** Tao He, Wei Fu, Jianqiao Xu, Zhihong Zhang, Jiuxing Zhou, Ying Yin, Zhenjie Xie

**Affiliations:** Department of Information Security, Naval University of Engineering, Wuhan, China

**Keywords:** neural network, interdisciplinary research, BERT, deep learning, vector space

## Abstract

Interdisciplinary research promotes the emergence of scientific innovation. Researchers want to find interdisciplinary research in their research field. However, the number of scientific papers published today is increasing, and completing this task by hand is time-consuming and laborious. A neural network is a machine learning model that simulates the connection mode of neurons in the human brain. It is an important application of bionics in the artificial intelligence field. This paper proposes an approach to discovering interdisciplinary research automatically. The method generates an IRD-BERT neural network model for discovering interdisciplinary research based on the pre-trained model BERT. IRD-BERT is used to simulate the domain knowledge of experts, and author keywords can be projected into vector space by this model. According to the keyword distribution in the vector space, keywords with semantic anomalies can be identified. Papers that use these author keywords are likely to be interdisciplinary research. This method is applied to discover interdisciplinary research in the deep learning research field, and its performance is better than that of similar methods.

## Introduction

Interdisciplinary research has promoted the emergence of scientific innovation and provides new growth points for the sustainable development of science. Scientific community and policy makers are concerned about interdisciplinary research (Van 2015). Many policies and funding initiatives have been designed to encourage interdisciplinary research (Huang et al., 2016). In this environment, researchers want to find interdisciplinary research in their research field. But researchers are usually only familiar with their own research fields and lack opportunities to communicate with researchers in other research fields. Besides, the number of scientific papers published today is increasing, and completing this task by hand is time-consuming and laborious. Therefore, an automatic method for discovering interdisciplinary research is necessary.

A neural network is a machine learning model that simulates the connection mode of neurons in the human brain. It is an important application of bionics in the artificial intelligence field and has been widely used in many fields (Jiang et al., 2019; Huang et al., 2021). A deep neural network is a neural network model with many layers. The advantage of this model is that it can automatically extract the required features from the original data according to the task requirements and abstract the extracted features layer by layer to obtain better model performance (Du et al., 2021; Jiang et al., 2021; Li et al., 2022). A small quantity of training data is used to fine-tune the pretrained deep neural network model parameters so that they can be suitable for specific tasks. Through this fine-tuning process, deep neural network model has achieved a series of remarkable research results in many fields, such as computer image recognition, video retrieval and natural language processing. In this paper, a deep neural network model, IRD-BERT, is generated to simulate the domain knowledge of experts by fine-tuning the parameters of the pre-trained model BERT (Vaswani et al., 2017; Devlin et al., 2018). Using IRD-BERT, interdisciplinary research can be automatically recognized from a large number of papers.

When researchers are looking for interdisciplinary research in their research field, a manual method is to first retrieve the papers in their research field from the literature database and then screen the author keywords of these papers to see whether there are some keywords that usually do not appear in their research field. Finally, the papers with these keywords are read to determine whether they are about interdisciplinary research. Because the number of papers in a research field is usually very large, it takes considerable time for researchers to find interdisciplinary research by this manual method. The manual method is also unable to achieve long-term automation monitoring of interdisciplinary research.

In this paper, automatic recognition of interdisciplinary research is realized by letting the computer simulate this manual method. Specifically, first, the pre-trained model BERT is fine-tuned by using a natural science literature corpus to generate a deep neural network model IRD-BERT, which is suitable for dealing with tasks related to scientific literature. Then, the author keywords in a certain research field are projected into vector space through IRD-BERT. The DBSCAN algorithm (Ester et al., 1996) is used to identify noisy sample points in the vector space, and the author keywords represented by these noisy sample points are often used in other research fields. Finally, the papers with such author keywords are given to the researchers to judge whether they are interdisciplinary research to achieve the discovery of interdisciplinary research. The method is applied to discover interdisciplinary research in the deep learning research field, and its performance is better than that of similar methods.

Compared with similar methods, this method improves the accuracy of interdisciplinary research discovery because of the rich semantic information of natural science in IRD-BERT. Additionally, it is not necessary to know in advance the specific discipline in which interdisciplinary research takes place; it also does not rely on the discipline of the journal in which the paper is published to define the discipline of the paper. It provides a novel solution to interdisciplinary research discovery.

## Related Works

Research on discovering interdisciplinary research can be divided into two categories. One category is retrospective research, which identifies and analyses the topics in papers when it is known that these papers are interdisciplinary (Wu et al., 2017; Dong et al., 2018). This kind of research is unable to address the problems that need to be solved in this paper. Another category is to find the papers engaged in interdisciplinary research from a number of papers, which is suitable for the problems to be solved in this paper.

Based on discovering interdisciplinary research, this category of research can be divided into citation-based methods, author-based methods and content-based methods. In the citation-based method, Porter et al. proposed an interdisciplinary measurement index based on the distribution of references in different disciplines (Porter et al., 2007); Mugabushaka et al. discussed the interdisciplinary index defined by the concept of biological diversity based on the discipline to which the citation belongs (Mugabushaka et al., 2016). In the author-based approach, Schummer et al. analyzed interdisciplinary developments in nanoscience and nanotechnology in terms of the institutions to which authors in the same paper belong (Schummer, 2004). Abramo et al. attempted to identify interdisciplinary research according to the specific classification of Italian scientists (Abramo et al., 2012). At present, a large number of studies focus on content-based methods. Xu et al. used TI and BET values to calculate the interdisciplinary attributes of subject words (Xu et al., 2016). The TI value reflects the number of disciplines associated with the subject words, while the BET value reflects the central degree of the subject words in the disciplines. Mao et al. quantitatively studied the knowledge diffusion model in interdisciplinary fields through knowledge memes, which overcomes the problem that the citation-based method only considers the number of citations but does not consider the real citation content (Mao et al., 2020). Kamada et al. proposed the diffusion meme index to evaluate the knowledge diffusion distance in the citation network, which can be used to discover interdisciplinary research (Kamada et al., 2021). In previous studies, many methods need to specify specific disciplines that produce interdisciplinarity in advance before they can identify interdisciplinary research, but when researchers do not know which disciplines intersect with their own research, it is difficult for them to work. There are also many studies that use the discipline of the journal where the paper is published to define the discipline of the paper. This method of defining the discipline of a paper is not accurate enough and may affect the effect of interdisciplinary research discovery.

Automation brings convenience to our lives (Liu X et al., 2022; Liu Y et al., 2022; Wu et al., 2022). It includes automatic image processing, automatic text processing and so on (Chen et al., 2022; Sun et al., 2022). The method proposed in this paper is the automated processing of text. In this paper, the author keywords of papers need to be expressed in the form of a digital vector. At present, this process is usually realized by neural network models. Bengio et al. proposed the method of generating vector representations of words using a neural network model (Bengio et al., 2003). He used a neural network to build a language model; after training the neural network, the corresponding vector representation of words can be extracted. Inspired by the method of Bengio, Word2Vec uses CBOW or Skip-gram to generate vector representations of words through neural networks (Mikolov, Chen, et al., 2013; Mikolov, Sutskever, et al., 2013). However, this method uses a vector to represent various semantics of words, which cannot solve the polysemy problem. To solve the polysemy problem, Peters et al. proposed ELMO, a vector representation of words related to context via neural networks (Peters et al., 2018). Then, the pre-trained models BERT (Vaswani, et al., 2017; Devlin, et al., 2018) and GPT (Radford et al., 2018) are proposed. In particular, fine-tuning the model parameters of BERT according to specific tasks to establish a new model suitable for the task, this neural network training method has achieved good results in many downstream tasks. In the scientific domain, based on BERT, a pretrained language model SciBERT was built; it is trained on a large corpus from the computer science and biomedical domain and has improved performance on downstream scientific NLP tasks (Beltagy et al., 2019). Next, Cohan et al. presented SPECTER, a model for learning representations of scientific papers, based on a transformer language model that is pretrained on citations (Cohan et al., 2020).

## IRD-BERT

This paper uses many natural science studies from various disciplines to fine-tune the parameters of BERT and generates a deep neural network model IRD-BERT (BERT for interdisciplinary research discovery). The model is used to represent author keywords in the form of digital vectors for discovering interdisciplinary research.

**FIGURE 1:**
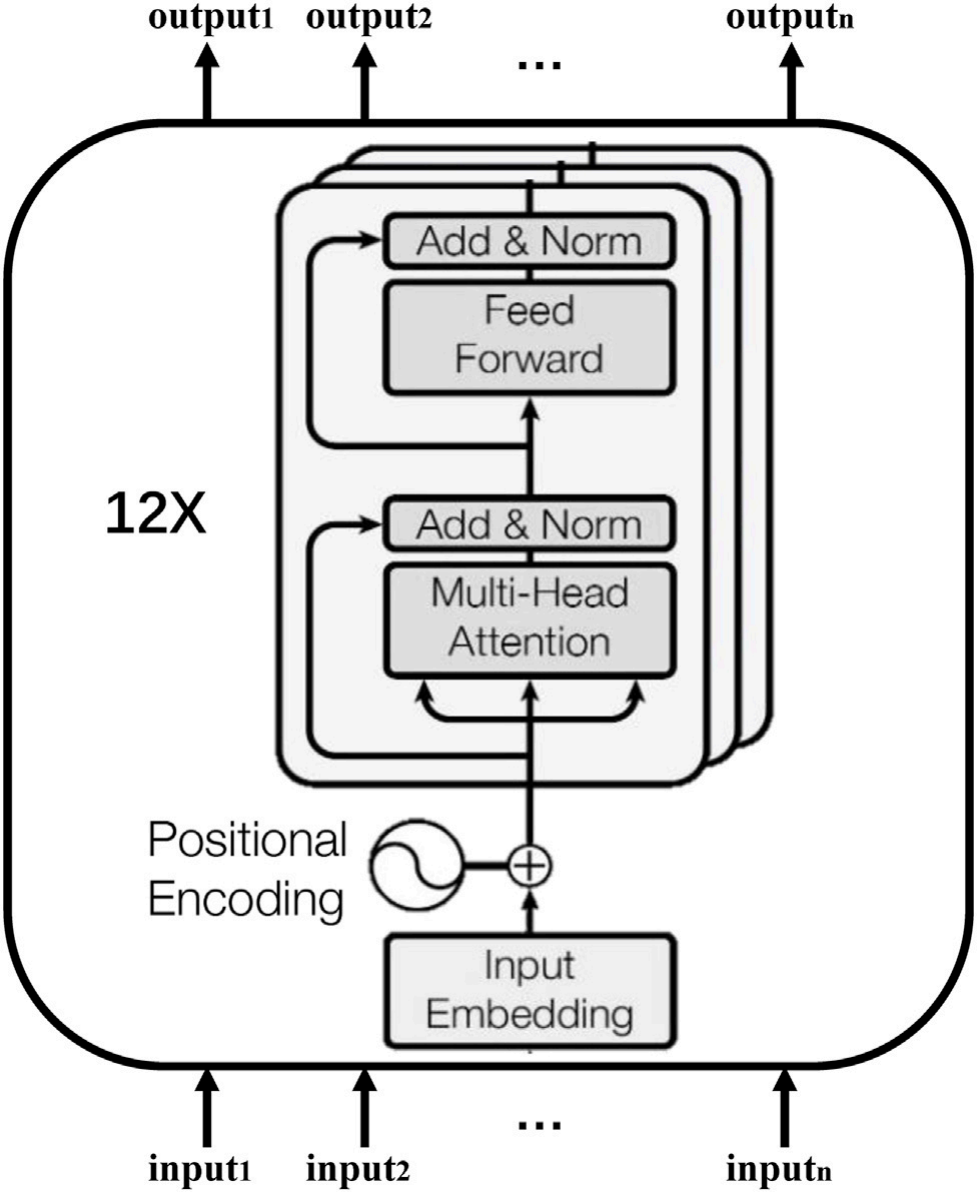
The BERT architecture.

BERT is a neural network pretrained model proposed by Google’s researchers in 2018. The structure of the base version of BERT is shown in Figure 1 (Vaswani, et al., 2017; Devlin, et al., 2018). It consists of 12 layers of bidirectional transformer encoders and has approximately 110 million trainable parameters. The multi-head attention in the encoder is helpful to extract useful features from the inputs. BERT was pretrained on the English Wikipedia corpus and Toronto Book corpus (Zhu et al., 2015) with two tasks: the masked language model (MLM) and next sentence prediction (NSP). During MLM, some of the tokens from the sequence were masked, and then correct tokens were predicted. For NSP, it connected two sentences from the corpus into a sequence and predicted whether it is reasonable for the latter sentence in the sequence to appear after the previous sentence.

In order to solve the problem raised in this paper, the author’s keywords need to be represented in the form of digital vectors, that can reflect the semantic information of natural science. However, the generated corpus of the BERT model is the Toronto Book corpus and English Wikipedia corpus, and most of the contents in these corpora come from public reading materials. Therefore, the model can reflect the common-sense semantic information, and it is difficult to accurately express the semantic information of author keywords from natural science. Although SciBERT (Beltagy, et al., 2019) can capture some semantic information of natural science, it only uses corpora from the computer science and biomedical domains in the fine-tuning process. Therefore, it is not suitable for our interdisciplinary research discovery task because interdisciplinary research discovery needs to use semantic information in various natural science research fields. Although SPECTER (Cohan, et al., 2020) can accurately convert scientific literature into the form of a digital vector, the semantic information contained in such vector representations is too rich, so it is difficult to identify interdisciplinary research in the vector space according to the distribution characteristics of vectors.

To solve this problem, this paper uses a certain scale of natural science literature covering a wide range of disciplines to fine-tune the parameters of BERT on the MLM task and generates the IRD-BERT model. Specifically, through the Web of Science service provided by Clarivate, approximately three million abstracts of SCI papers from 2013 to 2017 were collected. According to the Web of Science schema, the SCI papers published from 2013 to 2017 cover 152 research areas. The abstracts we used include all these research areas. Among them, the number of papers in the area of chemistry is the largest, with a number of about 380,000, and the number of papers in the area of dance is the smallest, only 258 papers. These abstracts are used to fine-tune the parameters of the base version of BERT model on the MLM task. We want to use IRD-BERT to discover interdisciplinary research related to deep learning in 2018. The abstracts of the papers in 2018 where the abnormal keywords are located may involve the context of deep learning research. If these abstracts are employed to fine-tune the BERT model to create the IRD-BERT model, then the vectors of abnormal keywords generated by IRD-BERT may not be far away from other keywords related to deep learning research in the vector space. Therefore, the abstracts after 2017 were not used. Besides, collecting abstracts is a heavy manual work. In the end, the abstracts from 2013 to 2017 were used for fine-tuning the BERT model. Whole word masking is used to enhance the effect of fine-tuning. Three training epochs are carried out on the corpus, and the IRD-BERT model is generated in 14 h on a workstation with dual NVIDIA GeForce RTX 3090.

Using IRD-BERT, author keywords can be represented as digital vectors. Before inputting the author keyword into IRD-BERT, they need to be expressed in the form of wordpiece (Schuster et al., 2012). After inputting a wordpiece segmentation into IRD-BERT, each layer of the model will have a corresponding vector output. According to previous studies (Reimers et al., 2019), the output from the second-to-last layer of the model is selected as the vector representation of the wordpiece segmentation. For author keywords composed of multiple wordpiece segmentations, the average of their vectors is used.

The vector representation of author keywords generated by IRD-BERT contains rich semantic information. Generally, in scientific literature, keywords with similar contexts are close to each other in vector space, and keywords with different contexts are far away in the vector space. According to the characteristics of this spatial distribution, an automatic method for discovering interdisciplinary research can be designed.

## Method of Discovering Interdisciplinary Research

The method of automatic discovery of interdisciplinary research proposed in this paper is a simulation of the process of manually identifying interdisciplinary research. When researchers want to find interdisciplinary research in their research field, they can first retrieve the relevant papers in the field from the academic database and then screen the author keywords in these papers one by one to see if there are any keywords that are quite different from the common words they are familiar with in this research field. Finally, the literature containing these keywords is read to determine whether they are interdisciplinary research. In this process, researchers need to screen the keywords in a large number of papers one by one according to their domain knowledge, which requires considerable time and effort. Using the spatial distribution characteristics of the author keyword vectors generated by IRD-BERT, we can simulate the domain knowledge of researchers and automatically identify interdisciplinary research in scientific literature.

**FIGURE 2:**
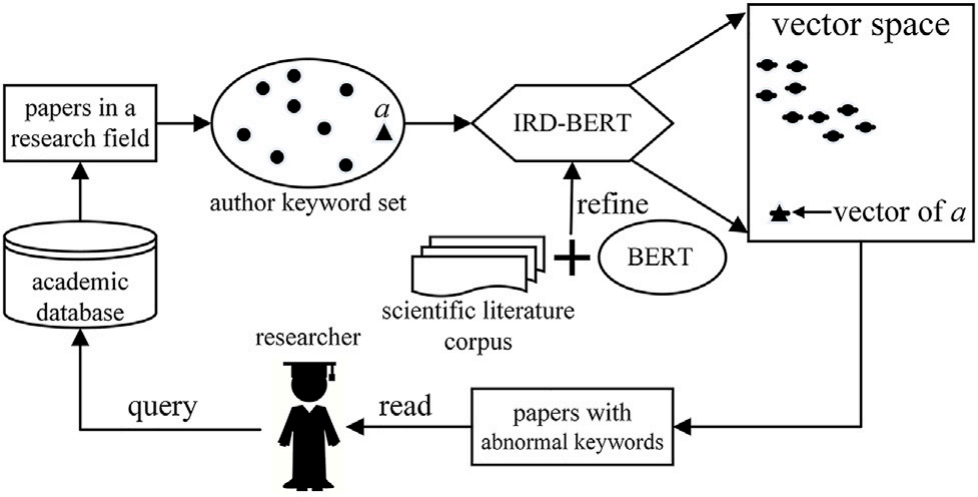
Discovering interdisciplinary research using IRD-BERT.

The process is shown in Figure 2. When discovering interdisciplinary research, first, the researcher submits a query to the academic database to retrieve the papers in their research field. Then, the computer extracts the author keywords from these papers, represents these keywords in the form of a digital vector through IRD-BERT, and uses the DBSCAN algorithm to identify the keyword which is far away from other keywords in the vector space. We call this type of keyword an abnormal keyword. Finally, the computer returns the papers containing these keywords to the researcher for reading to determine whether the literature is interdisciplinary research. In this process, the researcher only needs to complete the query submission and read a small number of returned papers; other work is performed automatically by the computer.

The process can be briefly described as follows:

**Input:** *q* (query for a certain field of research).
**Output:** *p* (interdisciplinary research papers to be confirmed).
1) Submit *q* to the databases and return some papers *m*.
2) For *author keyword* in *m*:
3) *Vector* of *keyword* = IRD-BERT (*author keyword*).
4) *Noisy vectors* = DBSCAN (*vectors of keywords*).
5) Map *noisy vectors* to its papers *p*.
6) Return *p*.

In this description, IRD-BERT stands for projecting an author keyword to a vector through the deep neural network model IRD-BERT, DBSCAN stands for identifying noisy vectors using the DBSCAN algorithm, and a noisy vector is a vector that stays far away from other vectors.

The principle of this method is explained below. The author keywords belonging to the same research field are similar in context, so their vectors are close to each other in vector space. If the vector representation of a keyword (such as author keyword *a* in Figure 2) is far away from other keywords in the vector space. This indicates that the context of this keyword is quite different from others. Then, this abnormal keyword is likely to be from papers in another discipline. According to this phenomenon, if we use the DBSCAN algorithm to identify noisy vectors in the vector space, we can find the abnormal keywords that are far away from other keyword vectors and then use them to discover interdisciplinary research. This is because if a point in the vector space is far away from other points, it will be identified as a noisy point by the DBSCAN algorithm.

## Discovery of Interdisciplinary Research in the Field of Deep Learning

In recent years, deep learning has become one of the important technologies leading the development of artificial intelligence. There are some studies that apply deep learning technology to various disciplines. According to the development characteristics of deep learning and combined with the suggestions of field experts, we took the natural science papers in the field of deep learning in 2018 as the experimental objects and use the proposed method to discover interdisciplinary research in these papers. Through discussion with five experts in the field, it was confirmed that in 2018, the main deep learning research fields included face recognition, speech recognition, medical image processing and so on. To discover interdisciplinary research in the deep learning research field, we first need to identify abnormal author keywords and then read the papers containing these author keywords to find the interdisciplinary research, which is described in detail one by one below.

### Abnormal Keyword Identification

With the help of domain experts, we retrieved 6,788 articles related to deep learning published in 2018 through the Web of Science service provided by Clarivate. All author keywords in these papers are extracted. To make the keywords more representative, we removed the keywords that only appeared once. If a word is polysemous, then the vector representation of this word generated by IRD-BERT may not be accurate enough. This is because the input of IRD-BERT is only author keyword, and one vector is used to represent multiple meanings of a keyword. In order to reduce the occurrence of this situation, the keywords composed of only one word were removed, because this kind of keyword is more likely to have multiple meanings. The remaining author keywords were used for subsequent experimental analysis. We have also tried to input the abstract of the article where keyword is located into IRD-BERT together with the keyword. This will make the vector representation of keywords contain certain context information. Different vectors are used to represent different meanings of the same word. However, the content of these abstracts is related to deep learning research, which may lead to the result that abnormal keyword will not be far away from other keywords in the vector space.

According to the principle of our method, when an author keyword comes from the research of other disciplines, its corresponding vector will be far away from the vectors of other keywords in the vector space. We chose to use the DBSCAN algorithm to identify these abnormal keywords. Since the DBSCAN algorithm cannot give the score of the abnormal degree of a point in the space, by adjusting the parameters of the DBSCAN algorithm, the 10 closest author keywords in the vector space and the 10 author keywords with the highest degrees of abnormality far away from other points in the vector space are obtained, as shown in Table 1.

**TABLE 1:** Top 10 author keywords with the highest and lowest degrees of abnormality.

	Author keywords
Highest degrees of abnormality	Bike sharing, DOA estimation, United Kingdom biobank, Remaining useful life, Time projection chambers, Porous media, Hot deformation, Fractal dimension, Design space exploration, Traditional Chinese medicine
Lowest degrees of abnormality	Convolutional network, Recurrent convolutional neural networks, Fully convolutional neural networks, Deep convolutional neural network, 3D convolutional neural network, Convolutional deep belief network, Convolutional neural networks, Fully convolutional networks, Convolutional neural network model, Piecewise convolutional neural networks

The table shows that the 10 nearest keywords in the vector space are the names of various convolutional neural networks and are common words in the deep learning research field. The keywords that are far away from other points in vector space are the keywords whose context is quite different from the context of the deep learning research field. We read the literature in which these keywords are located. Except for “design space exploration”, which comes from papers that use design space exploration technology to research the hardware of deep learning, the other nine keywords come from interdisciplinary research. For example, “time projection chambers” come from the papers that apply deep learning models to the high-energy physics field, and “traditional Chinese medicine” comes from the papers that apply deep learning techniques to the traditional Chinese medicine research field. These studies do not belong to the field of common research content of deep learning.

**FIGURE 3:**
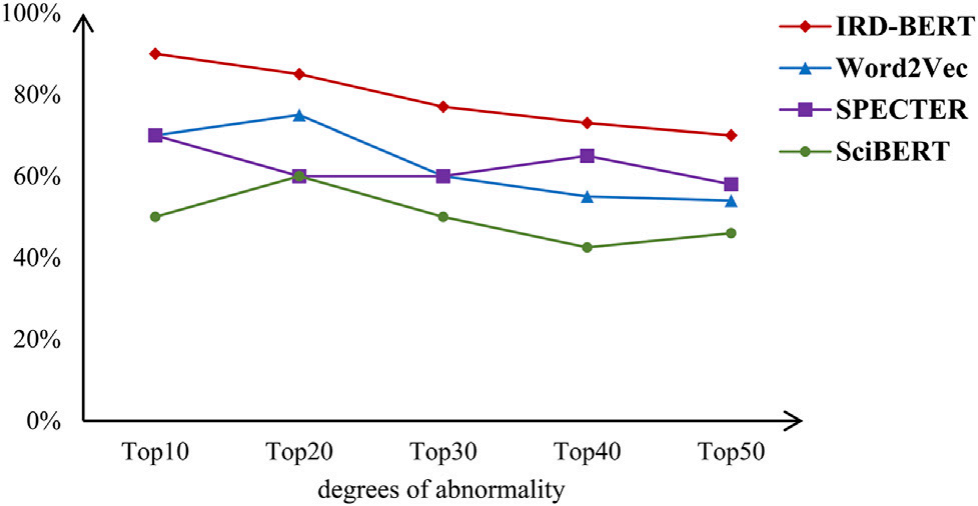
The proportion of vectors from interdisciplinary research.

The proposed method is compared with some similar methods. IRD-BERT and SciBERT were used to generate author keyword vectors, respectively. The previous Word2Vec-based method (He et al., 2021) was also used for projecting the author keywords to the vectors. In addition, we concatenated the title and the abstract of a paper to form a document, and input it into the SPECTER model to get the vector of this paper. After transforming the author keywords or the papers into vectors, the DBSCAN algorithm was employed for finding interdisciplinary research in the field of deep learning. Figure 3 shows the proportion of vectors which represent the author keywords or the papers with the most abnormal degree coming from interdisciplinary research. It can be seen in the figure that, for the problem raised in this paper, the method of using IRD-BERT is better than that of using Word2Vec, SPECTER, and SciBERT. Compared with the Word2Vec model, the IRD-BERT model use attention mechanism and deeper structure and is added with the common-sense semantic information from BERT. For the SPECTER-based method, compared with the semantic information contained in author keyword, the semantic information contained in title and abstract is too rich. This may make some interdisciplinary research papers are not far away from other papers in the vector space. The SciBERT model only uses corpora from the computer science and biomedical domains in the fine-tuning process. That’s probably not enough, because interdisciplinary research discovery needs to use semantic information in various science research fields. This may be the reason why it doesn’t work very well.

As seen in Figure 3, for our method, even the 10 author keywords with the highest degrees of abnormality do not all come from interdisciplinary research. We analyzed the data and found that the context of the identified abnormal words is different from deep learning papers, and these words are more common in other research fields, but there are still a small number of words in them used in deep learning research papers. For example, the context of the keyword “design space exploration” is more related to electronic system designs than to deep learning, so the vector of this word will be far away from the vectors of other keywords. However, in the deep learning literature we retrieved, this word comes from the paper on the hardware of deep learning, which makes the word unable to be used to discover interdisciplinary research. The decline in the IRD-BERT curve in Figure 3 is mostly caused by such author keywords, which reflects the complexity of language.

We have also analyzed the errors of the other methods. The SPECTER-based method uses the title and the abstract of a paper to represent the paper as a vector. The semantic information contained in the vector is too rich. Therefore, it is difficult to make a very specific analysis of the errors produced by this method. The errors produced by keyword-based method can be divided into two categories. One is that although some author keywords are identified as abnormal keywords because they seldom appear in the literature related to deep learning, they are used in deep learning research papers under certain circumstances. These keywords cannot be used to discover interdisciplinary research. For example, “design space exploration”, which has been mentioned before, is such an author keyword. The other is that some keywords are common in deep learning papers, but they are more widely used in other research fields, so they are identified as abnormal keywords in the vector space. We found that the majority of errors produced by the SciBERT-based method and the IRD-BERT-based method fell into the first category, but the majority of errors produced by the Word2Vec-based method fell into the second category. This phenomenon still needs to be further studied.

### Interdisciplinary Research in the Deep Learning Field

With the help of five experts in the field of deep learning, the papers using the top 50 author keywords with the highest degrees of abnormality were read. According to the development of deep learning in 2018, 35 interdisciplinary studies at that time were found among these papers. Limited by space, some of these researches are shown in Table 2. Deep learning involves interdisciplinary research in many fields, such as food, communications, and high-energy physics. the papers using the author keywords.

**TABLE 2:** Some interdisciplinary research discovered in deep learning papers.

Title	Abnormal keyword	Interdisciplinary research
Variety Identification of Single Rice Seed Using Hyperspectral Imaging Combined with Convolutional Neural Network	Rice seed	Apply deep learning to the field of food science
Remaining Useful Life Prediction for Lithium-Ion Battery: A Deep Learning Approach	Remaining useful life	Apply deep learning to the field of electronics
An Enhanced Deep Extreme Learning Machine for Integrated Modular Avionics Health State Estimation	Integrated modular avionics	Apply deep learning to the field of aviation
Deep Neural Networks for Energy and Position Reconstruction in EXO-200	Time projection chambers	Apply deep learning to the field of high energy physics
Deep Learning for Super-Resolution Channel Estimation and DOA Estimation Based Massive MIMO System	DOA estimation	Apply deep learning to the field of communication

## Conclusion

Neural network is a successful application of bionics in the artificial intelligence field. In this paper, the IRD-BERT model is generated by fine-tuning the parameters of the BERT deep neural network model with a scientific literature corpus. Then, IRD-BERT is used to project the author keywords into the vector space, and the distribution characteristics of the author keywords in the vector space are used to simulate the domain knowledge of experts to realize the automatic discovery of interdisciplinary research. Compared with traditional methods, this method makes full use of the semantic information of the author keywords in papers and has high accuracy in identifying the author keywords related to interdisciplinary research. Additionally, it does not need to know the specific discipline where interdisciplinary research takes place in advance, nor does it need to use the discipline of the journal where the paper is published to define the discipline of the paper.

When this method was verified in the field of deep learning, a problem was also found. Although the context of some author keywords is quite different from the deep learning research field, these keywords are used in the study of deep learning, not in interdisciplinary research. These author keywords contribute to the majority of errors in this method. It is not enough to rely on the semantic information of author keywords to solve the problem, additional information is needed. We will explore it in the future.

## Data Availability

The raw data supporting the conclusion of this article will be made available by the authors, without undue reservation.
